# Incidence Lung Cancer after a Negative CT Screening in the National Lung Screening Trial: Deep Learning-Based Detection of Missed Lung Cancers

**DOI:** 10.3390/jcm9123908

**Published:** 2020-12-02

**Authors:** Jungheum Cho, Jihang Kim, Kyong Joon Lee, Chang Mo Nam, Sung Hyun Yoon, Hwayoung Song, Junghoon Kim, Ye Ra Choi, Kyung Hee Lee, Kyung Won Lee

**Affiliations:** 1 Department of Radiology, Seoul National University Bundang Hospital, Seongnam-si 13620, Korea; jojoini05@gmail.com (J.C.); kjoon31@gmail.com (K.J.L.); whatisizstyle@gmail.com (S.H.Y.); souvient49@hanmail.net (H.S.); kimjhoon06@gmail.com (J.K.); kyung8404@gmail.com (K.H.L.); lkwrad@gmail.com (K.W.L.); 2 AI Research Group, Monitor Corporation, Seoul 06628, Korea; nam@monitorcorp.ai; 3 Department of Radiology, Seoul Metropolitan Government-Seoul National University Boramae Medical Center, Seoul 07061, Korea; cuteyera@gmail.com; 4 Department of Radiology, Seoul National University College of Medicine, Seoul National University Bundang Hospital, Seongnam-si 13620, Korea

**Keywords:** lung neoplasms, deep learning, computer-aided diagnosis, multidetector computed tomography, early detection of cancer

## Abstract

We aimed to analyse the CT examinations of the previous screening round (CT_prev_) in NLST participants with incidence lung cancer and evaluate the value of DL-CAD in detection of missed lung cancers. Thoracic radiologists reviewed CT_prev_ in participants with incidence lung cancer, and a DL-CAD analysed CT_prev_ according to NLST criteria and the lung CT screening reporting & data system (Lung-RADS) classification. We calculated patient-wise and lesion-wise sensitivities of the DL-CAD in detection of missed lung cancers. As per the NLST criteria, 88% (100/113) of CT_prev_ were positive and 74 of them had missed lung cancers. The DL-CAD reported 98% (98/100) of the positive screens as positive and detected 95% (70/74) of the missed lung cancers. As per the Lung-RADS classification, 82% (93/113) of CT_prev_ were positive and 60 of them had missed lung cancers. The DL-CAD reported 97% (90/93) of the positive screens as positive and detected 98% (59/60) of the missed lung cancers. The DL-CAD made false positive calls in 10.3% (27/263) of controls, with 0.16 false positive nodules per scan (41/263). In conclusion, the majority of CT_prev_ in participants with incidence lung cancers had missed lung cancers, and the DL-CAD detected them with high sensitivity and a limited false positive rate.

## 1. Introduction

The National Lung Screening Trial (NLST) demonstrated that three rounds of annual low-dose computed tomography (CT) screening (rounds T0, T1, and T2) reduced lung cancer mortality rates by 20% among asymptomatic high-risk participants [1]. Lung cancers in the NLST can be categorised into prevalent, interval, and incidence [2]. Prevalence of cancer refers to cancer detected on the first screening (T0), and interval cancer refers to cancer detected before the next scheduled screening (between T0 and T1 or between T1 and T2). Incidence cancer refers to cancer diagnosed through a scheduled incidence screening test, after a previous round of negative screening (detected through T1 or T2).

A previous study [3], which retrospectively analysed the CT examinations of 44 participants who were subsequently diagnosed with interval lung cancer, reported a higher number of false negative screens in the interval lung cancer group compared to the control group. Moreover, 73% (32/44) of interval lung cancers were in an advanced stage (stage III or IV), and 84% (37/44) of the patients died as a result of interval lung cancer. However, to the best of our knowledge, no previous study has investigated the previous round of CT screenings in participants who were subsequently diagnosed with incidence lung cancer. Liang et al. [4] reported that the computer-aided detection system (CAD) might be used as an effective second reader, which could detect up to 70% of the nodules missed by radiologists, and Ardila et al. [5] reported the possibility of end-to-end lung screening by means of the deep learning (DL)-based CAD.

Our study, therefore, aimed to retrospectively analyse the CT examinations of the previous screening round (CT_prev_) in the NLST participants diagnosed with incidence lung cancers and evaluate the value of DL-CAD in the detection of missed lung cancers.

## 2. Materials and Methods

We wrote this article adhering to the reporting guidelines [6,7,8].

### 2.1. Participants 

Consent to access NLST data was obtained from the National Cancer Data Access System (CDAS) of the National Cancer Institute (NCI), through a data transfer agreement between the authors and the NCI (project ID: NLST-379). This retrospective post hoc analysis used nonidentifiable participant data and was exempt from institutional review board approval at our institution (Institutional Review Board of Seoul National University Bundang Hospital, IRB exemption approval number: X-1906-544-903). The NLST was a randomized controlled trial involving 53,454 participants aged 55–74 years with a smoking history of at least 30 pack-years, including both current smokers and those who had quit within the past 15 years [9]. Participants were randomised for screening with either low-dose CT examinations or with posteroanterior chest radiographs three times annually at 33 screening centres across the USA and had follow-ups to evaluate the development of lung cancer and death.

In the NLST database [9], medical records were documented regarding diagnostic evaluation procedures for participants who had positive screening tests and for participants who were diagnosed with lung cancer. Tumour staging and pathology reports, records of operative procedures, and initial treatments were obtained for the participants with lung cancer.

### 2.2. Image Review 

We reviewed de-identified CT screenings of the NLST participants who were diagnosed with incidence lung cancer, defined as cancer diagnosed through incidence screening (rounds T1 and T2) after previous verifiable, negative CT screenings. We did not include the patients with positive results on the previous round of CT screening as they might be managed differently.

Two experienced radiologists (J.C. and Jihang Kim, with four and nine years of experience in the interpretation of CT examinations, respectively) independently classified CT_prev_ as positive or negative. The reviewers knew that the participants were diagnosed with incidence lung cancer through the next round of CT examinations, and they were otherwise blind to clinical information including age, sex, and current smoking status. Once we detected suspicious nodules on CT_prev_, we classified whether the nodules were positive or negative as per the NLST criteria and the lung screening and reporting & data system (Lung-RADS) classification [10], and reported them according to the lung cancer location recorded in the NLST database by anatomical site: lobe of lung, right or left hilum, or mediastinum. To confirm the presence of missed lung cancers, we referred to the NLST database [9] and the next round of CT examinations, where incidence lung cancers were detected. As we aimed to evaluate the accuracy of DL-CAD, the reviewers were asked to use enough time to detect missed lung cancers without omission. In case of discordant opinions, a third radiologist (K.W.L., with more than 20 years of experience in the interpretation of CT examinations) performed a thorough review of the images to reach final decisions, which were used as reference standards.

### 2.3. Diagnostic Criteria 

As per the NLST criteria [9], a positive low-dose CT screening test was defined as the detection of one or more indeterminate (noncalcified) nodules, measuring at least 4 mm in the longest diameter or, less commonly, lung consolidation or obstructive atelectasis, nodule enlargement, and nodules with suspicious changes in attenuation. As per the Lung-RADS classification [10], category 3 (probably benign) or higher corresponds to positive screening results, as determined by the bi-dimensional average diameter (mean nodule diameter of both the long and short axis) of the lesion. The criteria for the categorisation into Lung-RADS category 3 or higher include the detection of (1) a solid nodule of 6 mm or larger at the baseline or a new solid nodule of at least 4 mm, (2) a part-solid nodule of 6 mm or larger at the baseline or a new part-solid nodule of any size, or (3) a non-solid nodule of 30 mm or larger at the baseline or a new non-solid nodule of at least 30 mm.

### 2.4. Development of the DL-CAD 

The DL-CAD (LuCAS, Monitor Corporation, Seoul, Korea) employed a nodule detection algorithm, based on a three-dimensional (3D) convolutional neural network called DenseNet [11], running on a Linux operating system (Ubuntu 16·04, Canonical Ltd., London, UK), with CUDA^®^/CUDA^®^ deep neural network library (cuDNN) (versions 9.0 and 7.1, respectively, NVIDIA Corporation, Santa Clara, CA, USA) for graphic processing unit acceleration. The DL-CAD used the public Lung Image Database Consortium image collection (LIDC-IDRI) for training procedures. The LIDC-IDRI set provides a variety of information such as centre coordinates, boundaries, and appearance types for 1805 lung nodules included in 888 CT scans. The 1620 lung nodules contained in the 800 randomly chosen CT scans were used to train the neural network, while the 185 nodules in the remaining 88 CT scans were used for internal validation to obtain the optimal parameter with the maximum sum of sensitivity and specificity. The detection performance in the validation showed 0.95 sensitivity with 0.78 false positives per scan. Of note, all training (including internal validation) was completed on the LIDC-IDRI set. The NLST set was completely separated from training and was used only for evaluation. 

The DL-CAD algorithm also estimates nodule boundary and appearance type through other convolutional neural networks. Within the boundary, the algorithm can calculate the average diameter (size) of the nodule by finding the long and short diameters perpendicular to each other. The estimated size and the type determine the Lung-RADS category of the nodule. The performance of size and type estimation was reported in the clinical study approved by the Ministry of Food and Drug Safety (MFDS) of Korea (Protocol No. MO-PRT-LuCASP-KMLB). The average sizes of the total 97 nodules in the clinical study as measured by the reference standard (by radiologists) and the DL-CAD were 13.0 mm and 12.8 mm, respectively, showing no statistically significant difference (*p*-value = 0.6007). The consistency of type between the reference standard and the DL-CAD by the weighted Cohen’s kappa was 0.544 (95% confidence interval, 0.41, 0.68). 

### 2.5. Evaluation of the DL-CAD 

The DL-CAD reviewed CT_prev_ in participants diagnosed with incidence lung cancer (e.g., the DL-CAD reviewed the T1 screening CT of the patient diagnosed through the T2 screening). The analysed results were compared to the final decision adjudicated by the aforementioned radiologists to evaluate the performance of the DL-CAD. 

In order to evaluate the false positive (FP) rate of the DL-CAD, twofold controls were randomly selected among those CT screenings reported as negative by the NLST radiologists and not associated with a diagnosis of lung cancer during the trial. The positive results presented by the DL-CAD, after the analysis of the CT examinations in the control group, were independently reviewed by two radiologists (J.C. and Jihang Kim). In the case of a discordant opinion, a third radiologist (K.W.L.) made the decision, as previously mentioned.

### 2.6. Statistical Analysis 

Descriptive statistics were tabulated for the incidence lung cancer cases and for all lung cancer cases diagnosed in the NLST. We separately calculated patient-wise and lesion-wise sensitivities and the patient-wise FP rates of the DL-CAD in CT_prev_ of those participants diagnosed with incidence lung cancer, as per the NLST criteria and Lung-RADS classification. We also calculated the participant-wise rate of FP calls and the number of FP nodules per scan of the DL-CAD in the control group. 

## 3. Results

### 3.1. Demographics of the Study Cohort

**Figure 1:**
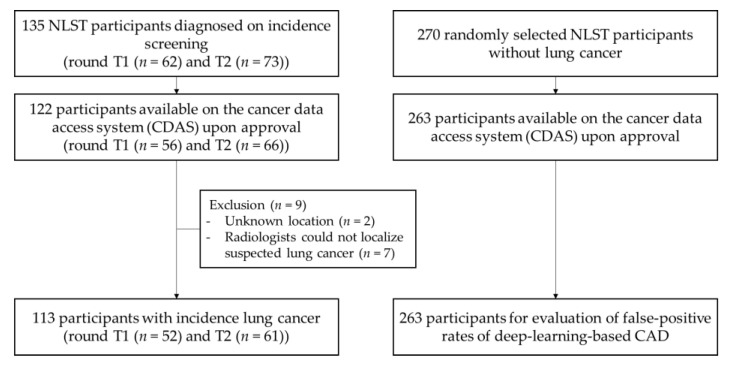
Participant flow diagram; NLST, National Lung Screening Trial; CAD, computer-aided diagnosis system.

In the NLST, 135 participants were diagnosed with incidence lung cancers after previous screenings with negative results. Among them, the CT images of 122 participants were available in the National Cancer Data Access System (CDAS), upon approval (Project ID, NLST-379) by National Cancer Institute (NCI) trial leadership; 56 and 66 incidence lung cancers were detected on the T1 and T2 CT screening rounds, respectively. Nine participants with incidence lung cancer were excluded from the study. Two were excluded because the anatomical site of the lung cancer was unspecified in the NLST database, and the other seven participants were excluded because the radiologists could not identify lung cancer candidates at the locations reported in the NLST database; the authors assumed that the positive results were observed in different anatomical sites and that there might be some errors in the database. The current study thus included 113 participants with incidence lung cancer (Figure 1). Of these, 41 were women (36%), 101 (89%) were non-Hispanic white, and 45 (40%) were former, rather than current, smokers. The median age was 63 years (range, 55–74 years), and the median smoking history was 60 pack-years (range, 30–168 pack-years). Cancers in the cohort were diagnosed at a median of 767 days from the NLST randomization (range, 360–1750 days). Of the 113 participants, 45 (40%) died of lung cancer during the follow-up period. The histopathologic characteristics of lung cancers, as recorded in the NLST database, are shown in Table 1.

### 3.2. Review of Previous-Round CT Screenings

As per the NLST criteria, 88% (100 of 113) of the CT_prev_ were positive and 74% of them (74 of 100 positive screens; 65%, 74 of 113 incidence lung cancers) had missed lung cancers at the corresponding locations recorded in the NLST database. As per the Lung-RADS classification, 82% (93 of 113) of the CT_prev_ were positive and 65% of them (60 of 93 positive screens; 53%, 60 of 113 incidence lung cancers) had missed lung cancers. We did not observe any significant differences in the stage, histologic subtype, size, location, and the mortality rates among the cases with missed lung cancers, including incidence lung cancers, and all screen-detected lung cancers (Table 1).

### 3.3. Diagnostic Performance of the DL-CAD

**Figure 2:**
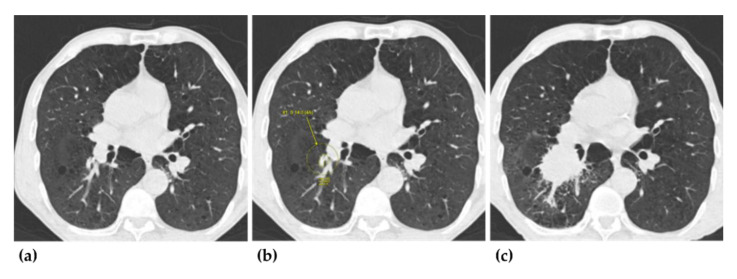
Missed lung cancer in the right lower lobe. (**a**,**b**) During the computed tomography (CT) examination of the first screening round (T0), the lesion was smaller in size and was missed by the interpreting radiologist (as shown in image a), but the deep-learning based computer-aided diagnosis system (DL-CAD) detected the missed lung cancer (as shown in image b) at the same round of CT screening. (**c**) The CT examination of the second screening round (T1) showed a large irregular mass, and the lesion was confirmed as lung cancer (adenocarcinoma).

**Figure 3:**
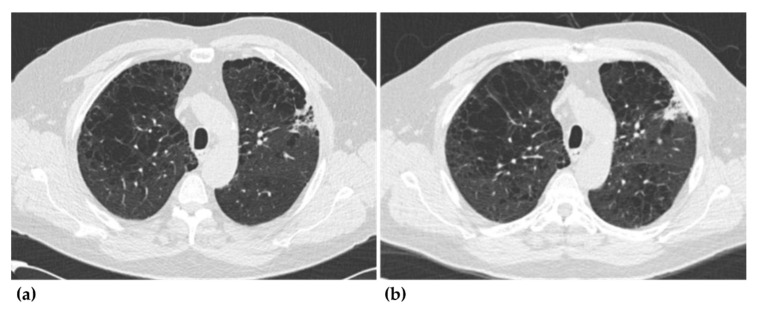
The only false-negative case reported by the deep-learning based computer-aided diagnosis system. On the CT examination of the first screening round, the lesion in the left upper lobe (as shown in image (**a**) was not detected by the radiologist involved in the national lung screening trial. The CT examination of the second screening round showed an enlarged consolidative mass (as shown in image (**b**), and the lesion was confirmed as lung cancer (adenocarcinoma).

As per the NLST criteria, the DL-CAD reported 98% (98 of 100; 95% confidence interval (CI), 93–99%) of the CT screenings as positive and detected 95% (70 of 74; 95% CI, 87–98%) (Figure 2) of the missed lung cancers. As per the Lung-RADS classification, the DL-CAD reported 97% (90 of 93; 95% CI, 91–99%) of the CT screenings as positive and detected 98% (59 of 60; 95% CI, 91–100%) (Figure 3) of the missed lung cancers. Patient-wise false positive (FP) rates were 15% (2 of 13; 95% CI, 4–42%) and 17% (4 of 23; 95% CI, 7–37%), as per the NLST criteria and the Lung-RADS classification, respectively.

Corresponding to the 135 participants diagnosed with incidence lung cancer in the NLST study, 270 participants with negative CT screenings were randomly selected as controls. Among them, 263 participants were included in the control group, whose CT images were available in the CDAS database. The DL-CAD reported 22.4% (59 of 263; 95% CI, 18–28%) of the CT screenings as positive, with 82 nodules of the Lung-RADS category 3 or higher. Among these, 32 CT screenings were concluded as true positive screens by the adjudicating radiologists, with 41 nodules of the category 3 or higher. The DL-CAD, therefore, recorded a FP call in 10.3% (27 of 263, 95% CI, 7–15%) of the control group, with 0.16 FP nodules per scan (41/263).

## 4. Discussion

The NLST resulted in 135 cases of incidence lung cancers, diagnosed after negative CT screenings, which was 0.37% of 36,929 negative CT screenings reported through the two screening rounds (T1 and T2). In line with previous studies [12,13], which reported that a majority of lung cancers could be retrospectively identified as nodules in the previous round of CT screenings, our study observed a positive consensus of interpretation in 82–88% of the CT_prev_ in participants diagnosed with incidence lung cancer. These findings may be attributed to the setting of a retrospective review, which involves an awareness of the potential presence of missed lung cancers. However, irrespective of these influences, the DL-CAD reported 95–98% of sensitivity in detection of the missed lung cancers, with limited FP results (10.3%) and FP nodules (0.16 nodules per scan). 

Compared to previous studies [4,14,15], which reported relatively low detection rates (56–78%) and many FP nodules (0.6 to 28 nodules per scan), the current study using computer-aided diagnosis CAD system developed with DL algorithm showed promising results. Unlike the NLST criteria, in which a noncalcified nodule of 4 mm or more is defined as a positive result [9], the Lung-RADS classification introduced at a later point is expected to reduce the FP rate [16], and The DL-CAD used in our study is also set to make a judgement based on this.

The plausible explanations for the failures in detecting lung cancers in CT screenings are the failures in detecting nodules and/or the underestimation of the detected nodules as non-malignant lesions that no longer need to be evaluated. The former is a detection error, while the latter is usually an interpretative error that occurs when the detected morphological structure of the lesion resembles a benign lesion, such as a scar or cyst [13]. Considering the clinical and histopathological characteristics of incidence lung cancers were not significantly different from all the other screen-detected cancers, the results of the current study suggest that the failure of previous screening rounds in detecting lung cancers could potentially be attributed to detection errors, rather than interpretation errors. In our study, the DL-CAD’s performance in the detection of pulmonary nodules showed the potential of minimising detection errors.

Low-dose chest CT screening considerably reduced lung cancer mortality rates [1,17], possibly due to an earlier diagnosis, when lung cancer is smaller and more curable. In the International Early Lung Cancer Action Program study [18], researchers found that 85% of screen-detected lung cancers were in the clinical stage I with a ten-year survival rate of 88%. According to the results of the current study, lung cancers could be missed in low-dose chest CTs screening, and the DL-CAD may facilitate early diagnosis and could potentially further reduce lung cancer mortality in lung cancer CT screening.

The reported number of negative CT screenings through T0 and T2 was 56,980 [1]. With simple extrapolation using the false positive rates of 12.2% (32/263), a maximum of 6933 negative scans would be relabelled to positive in the NLST. We may reduce the false positive rate using the Lung-RADS classification (27/263; 10.3%), and there would be a maximum of 5850 false negative CT scans with a reduction of more than 1000 CT scans. This should be noted when DL-CAD is widely utilised.

Our study has limitations. First, the interpretation of a CT screening in the NLST is subject to reader variability [19,20,21], even by experienced radiologists [22], and it is difficult to estimate the overall effect of less sensitive detection. Other undetected abnormalities may have met the criteria for positive screen in participants who did not develop incidence lung cancer. However, it is difficult to determine how the sensitive detection of positive results could affect the overall outcome of the NLST cohort. Second, the current study evaluated the performance of DL-CAD based on the detection of incidence lung cancers and not all lung cancers, which may cause a selection bias. Nevertheless, the detection of missed nodules in cases of incidence lung cancers, which are assumed to be smaller in size, is challenging and could be an indicator of the better performance of the DL-CAD. Third, the DL-CAD analysed CT images based only on one CT examination, which was the previous round of CT screening. Although the majority (125 of 135) of participants with incidence lung cancers had negative results on all previous screening rounds, 10 participants diagnosed with lung cancer in the third screening round (T2) had positive results on T0 and negative results on T1 screening. Therefore, it is possible that a small number of incidence lung cancers were diagnosed after interpretation errors rather than detection errors.

## 5. Conclusions

In this retrospective review of the NLST participants diagnosed with incidence lung cancer, the majority of CT examinations of the previous screening round met the criteria for a positive screen and had a missed lung cancer. The sensitivity of the DL-CAD was remarkable, with 95–98% in detection of missed lung cancers, while the FP rate was limited.

## Figures and Tables

**Table 1:** Clinical and histopathologic characteristics of lung cancers.

	Previously Missed Incidence Lung Cancer ^3^	All Incidence Lung Cancer	All Screen-Detected Lung Cancer
	**(*n* = 60)**	**(*n* = 113)**	**(*n* = 649)**
Stage ^1^			
IA	35/58 (60)	58/113 (51)	323/623 (51.8)
IB	5/58 (9)	6/113 (5)	61/623 (9.8)
IIA	8/58 (14)	16/113 (14)	45/623 (7.2)
IIB	1/58 (2)	2/113 (2)	20/623 (3.2)
IIIA	3/58 (5)	10/113 (9)	76/623 (12.2)
IIIB	2/58 (3)	5/113 (4)	17/623 (2.7)
IV	4/58 (7)	11/113 (10)	81/623 (13.0)
Histopathologic subtype			
Bronchioalveolar carcinoma	8/60 (13)	10/113 (9)	95/646 (14.7)
Adenocarcinoma	26/60 (43)	32/113 (28)	258/646 (39.9)
Squamous cell carcinoma	16/60 (27)	34/113 (30)	136/646 (21.1)
Large cell carcinoma	2/60 (3)	7/113 (6)	28/646 (4.3)
Non small cell carcinoma or other	6/60 (10)	16/113 (14)	75/646 (11.6)
Small cell carcinoma	1/60 (2)	12/113 (11)	49/646 (7.6)
Carcinoid	1/60 (2)	1/113 (1)	5/646 (0.8)
Size (median and interquartile range) ^2^	18 (12–22) mm	17 (11–25) mm	18 (12–28) mm
Location			
RUL	24/60 (40)	48/113 (43)	243/649 (38.5)
RML	3/60 (5)	6/113 (5)	44/649 (7.0)
RLL	9/60 (15)	16/113 (14)	104/649 (16.5)
LUL	16/60 (27)	28/113 (25)	158/649 (25.0)
LLL	9/60 (15)	13/113 (12)	89/649 (14.2)
Other	2/60 (3)	2/113 (2)	35/649 (5.3)
Mortality	16/60 (27)	45/113 (43)	225/649 (34.7)

Note—Data are n/N (%), unless otherwise indicated. ^1^ According to the seventh edition of the Cancer Staging Manual of the American Joint Committee on Cancer. ^2^ Lesion size of tumour on pathology. ^3^ According to the lung screening and reporting & data system (Lung-RADS) classification. RUL = right upper lobe. RML = right middle lobe. RLL = right lower lobe. LUL = left upper lobe. LLL = left lower lobe.
